# DIF in the Spanish Version of the Verbal Selective Reminding Test Using Samples From Hispanics in the United States, Mexicans, and Spaniards

**DOI:** 10.3389/fpsyg.2019.02687

**Published:** 2019-12-13

**Authors:** Manuel Morales-Ortiz, Fabiola Peña-Cardenas

**Affiliations:** ^1^Department of Experimental Psychology, University of Seville, Seville, Spain; ^2^Department of Psychology, Autonomous University of Tamaulipas, Matamoros, Mexico

**Keywords:** neuropsychology, memory, VSRT, Hispanic Americans, DIF

## Abstract

**Aim:**

Before a test can be used in the target population, it is necessary to demonstrate that there is measurement equivalence. One way to do this is by studying differential item functioning (DIF).

**Objective:**

In this study, we used the Mantel–Haenszel procedure and logistic regression to analyze DIF in the Spanish version of the Verbal Selective Reminding Test between Spaniards, Mexicans, and Hispanics in the United States.

**Method:**

Three balanced samples, matched by age, education, and sex, were studied: Spaniards, Mexicans, and Hispanics [616 healthy participants from Spain (*n* = 211), Mexico (*n* = 201), and the United States (*n* = 205)]. A six-trial version of the Spanish version of the Verbal Selective Reminding Test was administered and scored according to standard procedures.

**Results:**

Statistical analysis showed some DIF between the samples of Spaniards, Mexicans, and Hispanics. A bootstrap validation of results confirmed that the item *pollo* showed DIF: the Mexicans and Hispanics outperformed the Spaniards, holding a total score constant. The item *oído* also showed DIF and was remembered in greater measure by the Spaniards.

**Conclusion:**

The Spanish version of the Verbal Selective Reminding Test can be used with Hispanic populations.

## Introduction

In recent years the Hispanic/Latino population has been increasing, both in absolute terms and as a percentage of the total population of the United States. The most recent census data indicate that the figure is already over 59 million, which is more than 18% of the United States population (United States Census Bureau, 2017). This percentage is even higher if we include those who are unregistered, a population that has been estimated at 11.2 million (Puente et al., 2015). Most of this group come from Mexico, with smaller groups of other Latin-American populations (Puerto Ricans, Cubans, Central Americans, South Americans, and so on).

The neuropsychological assessment of this population in the United States poses a challenge for professionals as special training that takes multicultural aspects into account is necessary before the assessments can be successfully carried out (Uzzell et al., 2013; Salinas et al., 2016; Burke et al., 2019). For a long time, it was considered enough to use a test for English speakers that had been translated into Spanish. However, this strategy is not now considered suitable, even when it includes back translation (Salinas et al., 2016), since several studies have shown the effect of culture and ethnicity on the results of neuropsychological tests, with participants of non-English-speaking origin, and those of Spanish-speaking origin in particular, performing worst (Acevedo et al., 2000; Boone et al., 2007; Razani et al., 2007; Saez et al., 2014; Milman et al., 2018; Rosselli et al., 2019).

Most of the tests available for the English-speaking population in the United States cannot be considered suitable for the assessment of the Hispanic groups who live there (Elbulok-Charcape et al., 2014). The same thing applies when neuropsychological tests designed for Spaniards are used with Hispanic populations. Even though Spanish culture is closer to Hispanic populations than English-speaking culture, tests cannot be extrapolated directly from one culture to another. Studies are needed that support this kind of use and are able to detect possible bias in the evaluation of different cultures. Consequently, studies should be carried out to evaluate differential item functioning (DIF) in each of the cultures in which the test is going to be applied. This is a necessary prerequisite for the adaptation of a test (Pedraza and Mungas, 2008; Hambleton and Zenisky, 2011) and in order to consider the tests as conceptually equivalent (He and van de Vijver, 2012).

Various studies have revealed the differences found in the adaptation of the Mini Mental State Examination (MMSE) test. It has been found that the “no ifs, ands, or buts” item is usually easier for some Spanish-speaking populations (Ramirez et al., 2006), although this may be the result of bias in the adaptation of the test item in question. There are several Spanish language versions, and, whereas some of them translate the item by looking for linguistic equivalence, others seek to achieve the initial objective of the item, namely, the difficulty of repeatedly pronouncing consonant clusters (Ramirez et al., 2005). To give another example, Hispanics find it easier to recall English words that are similar to or the same as Spanish ones (such as the English “ranch” and “patio,” which are very similar to or the same as the Spanish “rancho” and “patio”). Similar results have been found in a good number of psychological tests that show ethnicity-related bias (Rosselli and Ardila, 2003; Rosselli et al., 2019).

Studies on DIF in neuropsychology with Spanish–American populations are extremely rare or non-existent. Only the MMSE has been studied in any depth, comparing Hispanic and English-speaking populations (Crane et al., 2006; Ramirez et al., 2006; Milman et al., 2018). DIF was found in various items, among which those associated with word recall deserve special mention (the repeat/recall item was easier for Latino participants). Ostrosky-Solis et al. (2007) compared different Spanish-speaking populations from Mexico, Colombia, and Spain and found that there were no cultural differences between those populations in a verbal fluency test. On the basis of these results, it is possible to consider the hypothesis that other tests developed with Spanish populations can also be used with Hispanic populations, provided that there are studies demonstrating that the tests are equivalent and that there is no DIF.

There are several methods for assessing DIF (Teresi, 2006; Magis et al., 2010). The main ones can be classified (Magis et al., 2011) according to (1) whether or not they are based on item-response theory (IRT) models and (2) whether they assess uniform DIF or non-uniform DIF. Uniform DIF occurs when the probability of a correct response in one item is greater for one group of subjects than another, holding ability being constant. Non-uniform DIF occurs when the probability of a correct response in one item for one group of subjects is greater for some levels of ability and lower for other levels of ability. They each have their advantages and drawbacks. The IRT method requires a large number of subjects and is based on some very restrictive assumptions, whereas methods not based on IRT can be used with fewer subjects and present a smaller number of assumptions (Finch, 2016). Various authors have pointed out that the results of DIF studies depend on the technique used, which is why they recommend using more than one technique to validate the results (Hambleton and Zenisky, 2011).

The present study applied methods that allow comparisons to be made between more than two groups and do not require excessively large sample sizes, namely the generalized Mantel–Haenszel (GMH) procedure and generalized logistic regression (GRL). The Mantel–Haenszel (MH) procedure is one of the most popular techniques for studying DIF because of its computational simplicity and its capacity for detecting DIF with small samples (Fidalgo and Scalon, 2010).

There are currently no studies indicating whether the Spanish version of the VSRT can be used with Hispanic populations. The objective of this study, therefore, was to examine whether DIF was present in the Spanish version of VSRT when applied to two Hispanic populations: Mexicans living in Mexico (*Mexicans*) and Mexicans who are resident in the United States (*Hispanics*). The hypothesis to be tested was that there was no DIF between the different populations.

## Materials and Methods

### Participants

Three balanced samples, matched by age, education, and sex, were studied: Spaniards, Mexicans, and Hispanics (descriptive statistics are presented in Table 1). The chi square statistic showed a similar proportion of men and women in the three groups [χ^2^(2, *N* = 617) = 0.33, *p* = 0.84]. There were no significant differences in age [*F*(2,572.02) = 0.835, *p* = 0.437] or educational level (number of years of formal school attendance [*F*(2,523.57) = 0.526, *p* = 0.591]. Subjects were excluded if they fulfilled any of the following criteria: (1) history of neuropathology; (2) hospitalization due to psychopathological disorders (e.g., schizophrenia, depression, etc.); (3) history of abnormal psychomotor development; (4) history of drug or alcohol abuse; (5) taking psychotropic medication that affects attention and concentration or causes sleepiness; (6) mother tongue was not Spanish. The three samples included some students. Researchers made an effort to obtain data from both rural and urban areas. The raw data are available upon request from the corresponding author.

**TABLE 1:** Samples distribution by age, education, and sex.

**Variable**	***N***	***Mean***	**Mdn**	***SD***	**TE**	**95% CI**		**Minimum**	**Maximum**
						**LL**	**UL**		
**Age**									
Spain	211	42.09	43	14.411	0.992	40.13	44.04	15	77
Mexico	201	43.77	45	18.327	1.293	41.22	46.32	15	79
United States	205	41.96	41	14.337	1.001	39.99	43.94	16	77
Total	617	42.59	43	15.767	0.635	41.35	43.84	15	79
**Education**									
Spain	211	11.25	11	3.635	0.250	10.75	11.74	2	21
Mexico	201	11.46	12	5.602	0.395	10.68	12.24	0	23
United States	202	11.00	12	4.095	0.288	10.43	11.57	0	21
Total	614	11.24	12	4.505	0.182	10.88	11.59	0	23

	**Gender**							
**Sample**	**Male**			**Female**			**Total**		
					
	**Frequency**	**Percent (%)**		**Frequency**	**Percent (%)**		**Frequency**		**Percent (%)**

Spain	62	27		149	71		211		100
Mexico	54	27		147	73		201		100
United States	57	28		148	72		205		100
Total	173	27		444	72		617		100

*Mdn, median; LL, lower limit; UL, upper limit.*

### Reference Group Sample: Spaniards

The data of participants for the reference sample were taken from 211 Spaniards, selected through non-probabilistic convenience sampling of study participants in a six-trial Spanish VSRT test suite to obtain normative data [see Morales et al. (2010) for a complete description of the sample]. Most participants were recruited in the south of Spain and these were matched by age, education, and sex with samples of the focal populations. Using the existing database, the procedure involved selecting the first subject who was of the same gender, approximately the same age (±1), and who had approximately the same number of years of education (±1) as the subjects studied in the Hispanic sample.

### Focal Group Sample: Mexicans

The sample of the first focal group (Mexicans) consisted of a total 201 adult and adolescent volunteers, selected in the city of H. Matamoros, Tamaulipas. The type of sample used was intentional, selecting those subjects who agreed to participate in the study and who met the same inclusion criteria as the sample of Spaniards but had Mexican nationality. The selection of the subjects was carried out by searching for the same percentage of men and women of similar ages and educational levels as the Hispanic sample.

### Focal Group Sample: Hispanics

The sample of the second focal group consisted of 205 adult and adolescent volunteers, selected in Brownsville, Texas. The sampling was intentional, selecting those subjects who agreed to participate in the study and who met the same inclusion criteria as in the previous samples. They also had to be of Mexican origin, live permanently in the United States, and have Spanish as their mother tongue. Table 1 shows the demographic data of the samples. 62.4% were from Mexico and the rest had been born in the United States, although their parents were originally from Mexico, 62.9% indicated that the language most used at home was Spanish.

### Materials

The Spanish version of VSRT form 1 was used to carry out this research (Campo et al., 2000; Campo and Morales, 2004). A questionnaire with personal details to identify the participants was also administered.

The VSRT is one of the most widely used to assess verbal learning and memory (Buschke and Fuld, 1974; Lezak et al., 2012). It has been used to investigate memory impairment in several different neuropathological and neuropsychiatric disorders. The validity and reliability of this test have been thoroughly studied with native English-speaking populations (Lezak et al., 2012). Different authors reported correlations between brain structures and different measures of SRT. So, Amato et al. (2008) reported that magnetic resonance imaging measures of temporal lobe volumes correlated with different SRT measures in multiple sclerosis patients. Zimmerman et al. (2008) also found that measures of verbal memory correlated with hippocampal volume.

A Spanish version of the VSRT has been developed and there is some evidence of its reliability and validity (Campo et al., 2000; Campo and Morales, 2004), with significant correlations between form 1 and form 2 that range between 0.65 and 0.72. Campo et al. (2003) also found differences between elderly healthy people and patients with dementia of the Alzheimer type using VSRT.

### Procedure

Spanish Form 1 consisted of 12 unrelated words: *Dado (Dice), Cinta (Ribbon), Norte (North), Jarro (Jug/Pitcher), Pollo (Chicken), Frente (Forehead), Llave (Key), Cruz (Cross), Fuego (Fire), Pena (Pity), Modelo (Model), Oído (Ear)*. After administering the six learning trials, multiple choice recognition trials were conducted. Twelve separate white index cards were presented to the subjects. Each card consisted of a list word and three foils (a phonemic foil, a semantic foil, and an unrelated foil). The participants were asked to identify the list word.

A single delayed-recall trial was given without prior warning 30 min after completion of the multiple-choice recognition trial. The interval was filled with other neuropsychological tests that did not involve memory. After the free delayed-recall trial, the multiple-choice recognition trial was conducted again. The six-trial version of the test was administered according to the procedure described by Buschke and Fuld (1974). The examiner presented the words at the rate of one word per 2 s. The entire list was read aloud to the subject only prior to the first recall trial. The subject was then asked to recall as many words from the list as possible and was subsequently reminded only of those words that s/he did not recall immediately preceding trial. For each trial, the subject’s responses were recorded. Intrusions were also recorded on each trial. The first time that a subject said a word that was not on the list, the examiner was allowed to say, “that word was not on the list.” The examiner was also allowed to ask the subject to run through the list out loud to make sure that s/he had not left anything out. As Buschke and Fuld (1974) pointed out, it was important to encourage the patient/subject to obtain the maximal retrieval on each recall trial. Words were not spelled or defined. The total number of words on the list was not disclosed to the subject. The procedure was continued until all 12 words were recalled on three consecutive trials, without any reminding, or until six trials had been exhausted. The test was scored according to the procedures described by Buschke and Fuld (1974). In this study we used only the sum of items recalled in each trial (TR), and only the first trial was analyzed to study DIF in order to avoid learning effects.

The tests were administered in private rooms with the least possible noise to distract individuals. The tests were performed individually with each subject and took 50–60 min on average to complete. The first activity in the evaluation process was to obtain the participants’ informed consent. A written form was devised that explained the general study objectives, procedures, implications for participants, and so on. The same consent form was given to each subject of the study to read carefully and, once concluded, any doubts that may have arisen during reading were clarified. Once read and signed, both the examiner and the assessed proceeded to the evaluation. For subjects under the age of 18, the informed consent of the parents or guardians was required. The ethics committee of the University approved the study.

### Data Analysis

Statistical analyses were performed with the R program v. 3.3.0 (R Core Team, 2018). The *difR* library was used for DIF analysis (Magis et al., 2010). The generalized Mantel–Haenszel (GMH) statistic and generalized logistic regression (GLR) were used due to the existence of multiple groups in the study design. The power to detect DIF items is usually lower than the power of a single test comparing all groups simultaneously (Penfield, 2001; Fidalgo and Madeira, 2008). The *difGMH*, *difGenLogistic*, *difMH*, and *difLogistic* functions were used with default values except for the purify option and *p*-value adjustment [the Benjamini–Hochberg (BH) method was chosen]. Lastly, model validation was performed using the *boot* and *boot.ci* function to calculate the confidence intervals for the statistical estimates. The *Nagelkerke* function of the *fmsb* library was used to calculate the *R*^2^ statistic for the logistic regression models.

## Results

Table 2 shows the percentages of correct answers in each sample and the results of the GMH statistic for each of the items along with the adjusted levels of significance. DIF (*p* adjusted < 0.05) was present in the items *dado, pollo, cruz, pena, modelo*, and *oído*.

**TABLE 2:** Differential item functioning comparisons between three groups using generalized Mantel–Haenszel (GMH), uniform DIF generalized logistic regression (GLRU), and non-uniform DIF generalized logistic regression (GLRNU) methods.

	**% CA**			**GMH**	**GLRU**			**GLRNU**		
				
**Item**	**Spaniard**	**Mexican**	**Hispanic**	**Statistic**	**Statistic**	**deltaR2**	**JG**	**Statistic**	**deltaR2**	**JG**
*Dado*	0.976	0.900	0.893	9.273^∗^	10.196^∗^	0.036	A	1.884	0.007	A
*Cinta*	0.635	0.577	0.493	3.754	4.615	0.008	A	1.274	0.003	A
*Norte*	0.232	0.284	0.259	2.631	2.539	0.005	A	0.139	0.000	A
*Jarro*	0.275	0.259	0.273	1.316	1.148	0.002	A	1.335	0.003	A
*Pollo*	0.275	0.353	0.380	9.141^∗^	7.718^∗^	0.015	A	5.056	0.011	A
*Frente*	0.389	0.363	0.293	2.650	2.349	0.005	A	0.125	0.000	A
*Llave*	0.289	0.338	0.322	2.106	2.071	0.004	A	1.919	0.004	A
*Cruz*	0.436	0.308	0.278	7.538^∗^	8.260^∗^	0.014	A	1.103	0.002	A
*Fuego*	0.275	0.289	0.249	0.064	0.072	0.000	A	7.851	0.017	A
*Pena*	0.502	0.299	0.278	17.203^∗∗^	19.114^∗∗^	0.033	A	0.082	0.000	A
*Modelo*	0.327	0.199	0.268	8.977	8.683^∗^	0.016	A	0.279	0.001	A
*Oído*	0.635	0.348	0.341	30.240^∗∗^	33.470^∗∗^	0.058	B	0.891	0.002	A

*JG, Jodoin and Gierl effect size criteria; % CA, correct answer percentage; ^∗^*p* < 0.05; ^∗∗^*p* < 0.01.*

Table 3 shows the results of the comparison between the different samples using the MH procedure. With reference to the samples of Spaniards and Mexicans, only the item *pollo* showed significant DIF when only *p*-values were considered. However, using ETS criteria based on effect size, two items appeared with a high DIF score: *dado* and *pollo.* Gómez-Benito et al. (2009) have pointed out that the combination of the criteria of significance and effect size reduces the rate of false positives in DIF studies. By applying these criteria, only important DIF appeared in the item *pollo*.

**TABLE 3:** Differential item functioning comparisons between samples using Mantel–Haenszel test (MH).

	**Spaniards/Mexicans**					**Spaniards/Hispanics**					**Mexicans/Hispanics**				
			
**Item**	**logOR**	**Alpha**	***p*.Adj**	**Delta**	**ETS**	**logOR**	**Alpha**	***p*.Adj**	**Delta**	**ETS**	**logOR**	**Alpha**	***p*.Adj**	**DeltaMH**	**ETS**
*Dado*	−1.955	0.319	0.101	2.684	C	−0.609	0.658	0.586	2.684	A	−0.106	0.962	0.915	0.090	A
*Pollo*	3.830	2.439	0.001	2.095	C	4.086	2.732	0.000	2.095	C	0.496	1.115	0.915	0.256	A
*Cruz*	1.314	1.444	0.283	0.863	A	1.101	1.332	0.406	0.863	A	−0.975	0.779	0.812	0.586	A
*Pena*	−0.508	0.871	0.712	0.324	A	0.545	1.178	0.586	0.324	A	−0.831	0.806	0.812	0.508	A
*Modelo*	0.370	1.118	0.712	0.262	A	2.498	1.956	0.038	0.262	C	1.670	1.575	0.570	1.067	B
*Oído*	−2.051	0.596	0.101	1.215	B	−1.418	0.680	0.312	1.215	A	−0.273	0.938	0.915	0.150	A

*logOR, logarithm odds ratio; Alpha, Alpha MH; *p*.Adj, *p*-value Benjamini–Hochberg; Delta, Delta MH; ETS, Educational Testing Service effect size criteria.*

After comparing Spaniards with Hispanics, the items *pollo* and *modelo* displayed significant DIF, which also showed fairly large DIF using the ETS criteria. It is interesting to note that the item *pollo* was used more frequently by the Hispanic population than by Spaniards.

When the results of the Mexican and Hispanic samples were compared, significant DIF was not present in any item. Only the item *modelo* showed moderate DIF using ETS criteria.

### GLR Procedure

Using this method to test uniform DIF (see Table 2), and following the criterion of statistical significance, it was found that the items *dado, pollo, cruz, pena, modelo*, and *oído* also displayed DIF. Nevertheless, when the effect size criterion (Nagelkerke’s *R*^2^) proposed by Jodoin and Gierl (2001) was used, only one item (*oído*) presented moderate DIF. This criterion is the one recommended in the literature (Gómez-Benito et al., 2009). The results of the non-uniform DIF test did not show this type of test bias (see Table 2). No item presented significant *p*-values, and effect sizes were low.

It can be stated, in conclusion, that when the criteria of significance and effect size were considered together, both the MH and LR procedures coincided in that the majority of the items did not display DIF and exhibited only a few discrepancies. When the samples of Spaniards and Mexicans were compared, discrepancies were found for the item *pollo*, which displayed DIF using the MH procedure, and the item *oído*, which was marked as DIF using logistic regression. In the comparison between the samples of Spaniards and Hispanics, discrepancies were found between the MH and LR procedures for the items *pollo*, *modelo*, and *oído*.

### Validation of the Models

In order to try and resolve the discrepancy between the MH and GLR procedures, we decided to validate them using the *bootstrap* method. The R package *boot* function simulated 1,000 iterations of the MH statistic and the regression method. Table 4 shows the results of the simulation. The number of tests that reported a significant result (*p* < 0.05) and the degree of DIF according to the effect size criterion (A, negligible; B, moderate; C, large) were recorded. DIF was considered to occur when there were significant differences (*p* < 0.05) and an effect size of B or C.

**TABLE 4:** Results of the validation study using bootstrap and effect size criterion.

	**Spaniards/Mexicans**						**Spaniards/Hispanics**					
		
		**MH**			**LR**			**MH**			**LR**	
								
		**NS**	**S**		**NS**	**S**		**NS**	**S**		**NS**	**S**
*Pollo*	A	480	2	A	0	879	A	168	1	A	0	578
	B	26	313	B	28	86	B	30	339	B	139	245
	C	0	179	C	7	0	C	0	463	C	38	0

*Modelo*							A	914	0	A	0	995
							B	47	37	B	0	4
							C	0	2	C	1	0

*Oido*	A	0	0	A	0	322	A	168	0	A	0	80
	B	0	9	B	201	202	B	30	339	B	280	152
	C	0	991	C	275	0	C	0	463	C	488	0

*A, negligible; B, moderate; C, large. NS, not significant (*p* ≥ 0.05); S, significant (*p* < 0.05); MH, Mantel–Haenszel test; LR, logistic regression.*

The results obtained confirmed that the methods showed discrepancies for the occurrence of DIF in two items: *pollo* and *oído.* Nevertheless, neither of the two methods showed DIF for the item *modelo* (only 3.9% of simulations found significant differences and DIF B using the MH technique and 0.4% using GLR).

## Discussion

Neuropsychological assessments in different countries generally makes use of tests that have been standardized for other populations. It is common practice, therefore, to use neuropsychological tests devised for native English speakers and translated into other languages in order to evaluate populations of Hispanic origin (Elbulok-Charcape et al., 2014; Salinas et al., 2016). This is the case in Mexico and Spain, and it enables considerable cost saving to be made and makes the work of neuropsychology professionals easier. Nonetheless, it has been obvious on a number of occasions that this procedure is inappropriate for performing validated neuropsychological evaluations (Meredith and Teresi, 2006; Hambleton and Zenisky, 2011). Various studies have shown that Spanish speakers are more likely to be categorized as “impaired” despite being clinically evaluated as normal (Ramirez et al., 2006; Siedlecki et al., 2010; Rosselli et al., 2019). Consequently, it is necessary to take cultural differences between the reference and focal populations into account. If the same test is to be used, DIF studies should be performed to ensure that the evaluation is fair.

In the present study, we evaluated the possibility of using form 1 of the Spanish version of the VSRT on Mexican and Hispanic populations, since we already have normative and validation studies of the test on Spaniards. The results show that there were very few differences between the responses of the Mexican and Hispanic populations. This can be interpreted as the Hispanic populations of Mexican origin maintaining their cultural references in spite of being immersed in a different culture. The first conclusion that can be drawn from our study, therefore, is that the normative data of Mexican populations may be valid for the neuropsychological evaluation of Hispanics resident in the United States, at least for the verbal memory test used here and for Hispanic populations of Mexican origin. In future studies, it would be interesting to determine whether these conclusions can be extrapolated to other Hispanic American populations resident in the United States and in their countries of origin. It should be pointed out that, at least for the evaluation of verbal memory, there is a test available that allows the neuropsychologist to evaluate clients of Hispanic origin with greater reliability and increased diagnostic sensitivity. Using tests that do not meet these requirements implies obtaining measurements that do not represent the true ability of the individual.

The results also showed some differences between the Mexican and Hispanic populations versus the population from Spain when the MH test was used. When the samples of Spaniards and Mexicans were compared, significant DIF was found for the item *pollo* and for *pollo/modelo* when the samples of Spaniards and Hispanics were compared. For the item *pollo*, the percentage of responses was favorable for the Mexican and Hispanic populations. This could be explained as a greater presence of this item at the cultural level in these populations and is consistent with the result indicated earlier that the two populations share the same cultural referents. Nevertheless, the same results were not found using the LR test or in the validation study. Various authors have pointed out that the MH technique tends to increase the percentage of false positives (Gómez-Benito and Navas-Ara, 2000; Penfield, 2001; Fidalgo and Scalon, 2010; Finch, 2016).

Likewise, for the item *oído*, moderate DIF was only present with the LR technique and occurred in only 15% of cases in the validation study. On the other hand, the validation study did show large DIF for this item with the MH technique. Consequently, there were two items (*pollo* and *oído*) for which the validation study did not present conclusive results. In the case of the MH technique, the most plausible conclusion is the presence of DIF (in favor of the Mexican and Hispanic populations in the case of the item *pollo*, and vice versa in the case of the item *oído*). If the results obtained with the LR method are taken into consideration, the conclusion would favor the absence of large DIF for these items. Nonetheless, considering the worst case (presence of DIF), the test remains valid for studying Mexican and/or Hispanic populations since the two items that display DIF have a compensatory effect. In one case it benefits the population of Spaniards, and in the other the Mexican or Hispanic populations. Some authors have contemplated the possibility of keeping items that present DIF in the test when there may be a compensatory effect between the groups, as in this case (Steinberg and Thissen, 2006).

It may be concluded, therefore, that the evidence of a high degree of DIF when comparing populations of Spaniards, Mexicans, and Hispanic Americans is inconclusive and that any possible DIF has a compensatory effect. The normative data obtained for the population of Spaniards (Morales et al., 2010) can be considered for use on these populations, enabling evaluations of verbal memory to be carried out with a minimum assurance of being able to objectively identify whether or not there is a possible deficit in these populations.

Nevertheless, these conclusions should be interpreted with caution since the sample sizes used make it impossible to detect items that display low or moderate DIF when the MH technique is used. Future research should be directed at studying further the existence of DIF between these and other Latin-American populations by increasing the size of the samples. Similarly, it would be interesting to expand the range of populations to include individuals from other South American countries.

## Data Availability Statement

The datasets generated for this study are available on request to the corresponding author.

## Ethics Statement

The studies involving human participants were reviewed and approved by the University of Seville. Written informed consent to participate in this study was provided by the participants’ and their legal guardian/next of kin where appropriate.

## Author Contributions

MM-O contributed to the conception and design of the study, performed the statistical analysis, and wrote the first draft of the manuscript. FP-C collected data, organized the database, contributed to the manuscript revision, and read and approved the submitted version.

## Conflict of Interest

The authors declare that the research was conducted in the absence of any commercial or financial relationships that could be construed as a potential conflict of interest.

## References

[B1] Acevedo A. Loewenstein D. A. Barker W. W. Harwood D. G. Luis C. Bravo M. (2000). Category fluency test: normative data for English- and Spanish-speaking elderly. *J. Int.Neuropsychol. Soc.* 6 760–769. 10.1017/s1355617700677032 11105466

[B2] Amato M. P. Zipoli V. Portaccio E. (2008). Cognitive changes in multiple sclerosis. *Expert Rev. Neurother.* 8 1585–1596. 10.1586/14737175.8.10.1585 18928350

[B3] Boone K. Victor T. Wen J. Razani J. Ponton M. (2007). The association between neuropsychological scores and ethnicity, language, and acculturation variables in a large patient population. *Arch. Clin. Neuropsychol.* 22 355–365. 10.1016/j.acn.2007.01.010 17320344

[B4] Burke S. L. Naseh M. Rodriguez M. J. Burgess A. Loewenstein D. (2019). Dementia-related neuropsychological testing considerations in non-hispanic white and latino/hispanic populations. *Psychol. Neurosci.* 12 144–168. 10.1037/pne0000163 31649798 PMC6812579

[B5] Buschke H. Fuld P. (1974). Evaluating storage, retention, and retrieval in disordered memory and learning. *Neurology* 24 1019–1025. 10.1212/WNL.24.11.1019 4473151

[B6] Campo P. Morales M. (2004). Normative data and reliability for a Spanish version of the verbal selective reminding test. *Arch. Clin. Neuropsychol.* 19 421–435. 10.1016/S0887-6177(03)00075-1 15033226

[B7] Campo P. Morales M. Juan-Malpartida M. (2000). Development of two Spanish versions of the verbal selective reminding test. *J. Clin. Exp. Neuropsychol.* 22 279–285. 10.1076/1380-3395(200004)22:2;1-1;ft279 10779841

[B8] Campo P. Morales M. Martínez-Castillo E. (2003). Discrimination of normal from demented elderly on a Spanish version of the verbal selective reminding.test. *J. Clin. Exp. Neuropsychol.* 25 991–999. 10.1076/jcen.25.7.991.16492 13680445

[B9] Crane P. K. Gibbons L. E. Jolley L. Belle G. Van Selleri R. Dalmonte E. (2006). Differential item functioning related to education and age in the Italian version of the mini-mental state examination. *Int. Psychoger.* 18 505–515. 10.1017/S1041610205002978 16478571

[B10] Elbulok-Charcape M. M. Rabin L. A. Spadaccini A. T. Barr W. B. (2014). Trends in the neuropsychological assessment of ethnic/racial minorities: a survey of clinical neuropsychologists in the United States and Canada. *Cultur. Divers. Ethnic Minor. Psychol.* 20 353–361. 10.1037/a0035023 25045947

[B11] Fidalgo A. M. Madeira J. M. (2008). Generalized Mantel-Haenszel Methods for Differential Item Functioning Detection. *Educ. Psychol. Meas.* 68 940–958. 10.1177/0013164408315265

[B12] Fidalgo A. M. Scalon J. D. (2010). Using generalized mantel-haenszel statistics to assess DIF among multiple groups. *J. Psychoeduc. Assess.* 28 60–69. 10.1177/0734282909337302

[B13] Finch W. H. (2016). Detection of differential item functioning for more than two groups: a monte carlo comparison of methods. *Appl. Meas. Educ.* 29 30–45. 10.1080/08957347.2015.1102916

[B14] Gómez-Benito J. Hidalgo M. D. Padilla J. L. (2009). Efficacy of effect size measures in logistic regression: an application for detecting DIF. *Methodology* 5 18–25. 10.1027/1614-2241.5.1.18

[B15] Gómez-Benito J. Navas-Ara M. J. (2000). A comparison of chi-squared. RFA and IRT based procedures in the detection of DIF. *Qual. Quant.* 34 17–31.

[B16] Hambleton R. K. Zenisky A. L. (2011). “Translating and adapting tests for cross-cultural assessments,” in *Culture and Psychology. Cross-Cultural Research Methods in Psychology*, eds Matsumoto D. van de Vijver F. J. R. (New York, NY: Cambridge University Press), 46–74.

[B17] He J. van de Vijver F. (2012). Bias and equivalence in cross-cultural research. *Online Read. Psychol. Cult.* 2 1–19. 10.9707/2307-0919.1111

[B18] Jodoin M. G. Gierl M. J. (2001). Evaluating type I error and power rates using an effect size measure with the logistic regression procedure for DIF detection. *Appl. Meas. Educ.* 14 329–349. 10.1207/S15324818AME1404_2

[B19] Lezak M. D. Howieson D. B. Bigler E. D. Tranel D. (2012). *Neuropsychological Assessment.* Oxford: Oxford University Press.

[B20] Magis D. Béland S. Tuerlinckx F. De Boeck P. (2010). A general framework and an R package for the detection of dichotomous differential item functioning. *Behav. Res. Methods* 42 847–862. 10.3758/BRM.42.3.847 20805607

[B21] Magis D. Raîche G. Béland S. Gérard P. (2011). A Generalized logistic regression procedure to detect differential item functioning among multiple groups. *Int. J. Test.* 11 365–386. 10.1080/15305058.2011.602810 31732280

[B22] Meredith W. Teresi J. A. (2006). An essay on measurement and factorial invariance. *Med. Care* 44(Suppl. 3), S69–S77. 10.1097/01.mlr.0000245438.73837.89 17060838

[B23] Milman L. H. Faroki-Sha Y. Corcoran C. D. Damele D. M. (2018). Interpreting mini-mental state examination performance in highly proficient bilingual bilingual Spanish-English and Asian Indian-English speakers: demographic adjustments, item analyses, and supplemental measures. *Am. J. Speech Lang. Pathol.* 27 975–987. 10.1044/2018_AJSLP-17-0074 29486488

[B24] Morales M. Campo P. Fernández A. Moreno D. Yañez J. Sañudo I. (2010). Normative data for a six-trial administration of a Spanish version of the verbal selective reminding test. *Arch. Clin. Neuropsychol.* 25 745–761. 10.1093/arclin/acq076 20978022

[B25] Ostrosky-Solis F. Gutierrez A. Flores M. Ardila A. (2007). Same or different? Semantic verbal fluency across Spanish-speakers from different countries. *Arch. Clin. Neuropsychol.* 22 367–377. 10.1016/j.acn.2007.01.011 17296282

[B26] Pedraza O. Mungas D. (2008). Measurement in cross-cultural neuropsychology. *Neuropsychol. Rev.* 18 184–193. 10.1007/s11065-008-9067-9 18814034 PMC2925437

[B27] Penfield R. D. (2001). Assessing differential item functioning among multiple groups: a comparison of three mantel-haenszel procedures. *Appl. Meas. Educ.* 14 235–259. 10.1207/S15324818AME1403_3

[B28] Puente A. E. Ojeda C. Zink D. Portillo-Reyes V. (2015). “Neuropsychological testing of Spanish speakers,” in *Psychological Testing of Hispanics: Clinical, Cultural, and Intellectual Issues*, 2nd Edn, ed. Geisinger K. F. (Washington: American Psychological Association), 135–152. 10.1037/14668-008

[B29] R Core Team (2018). *R: A Language and Environment for Statistical Computing.* Vienna: R Core Team.

[B30] Ramirez M. Ford M. E. Stewart A. L. Teresi J. (2005). Measurement issues in health disparities research. *Health Serv. Res.* 40 1640–1657. 10.1111/j.1475-6773.2005.00450.x 16179000 PMC1361222

[B31] Ramirez M. Teresi J. A. Holmes D. Gurland B. Lantigua R. (2006). Differential item functioning (DIF) and the mini-mental state examination (MMSE). *Med. Care* 44(Suppl. 3), S95–S106. 10.1097/01.mlr.0000245181.96133.db 17060840

[B32] Razani J. Murcia G. Tabares J. Wong J. (2007). The effects of culture on WASI test performance in ethnically diverse individuals. *Clin. Neuropsychol.* 21 776–788. 10.1080/13854040701437481 17676543

[B33] Rosselli M. Ardila A. (2003). The impact of culture and education on non-verbal neuropsychological measurements: a critical review the impact of culture and education on non-verbal neuropsychological measurements: a critical review, *2626*(October 2017). *Brain Cogn.* 52 326–333. 10.1016/S0278-2626(03)00170-2 12907177

[B34] Rosselli M. Tappen R. M. Newman D. (2019). Semantic interference test: evidence for culture and education fairness from an ethnically diverse sample in the USA, *34*(April 2018), 337–349. *Arch. Clin. Neuropsychol.* 34 337–349. 10.1093/arclin/acy037 29688251

[B35] Saez P. A. Bender H. A. Barr W. B. Rivera Mindt M. Morrison C. E. Hassenstab J. (2014). The impact of education and acculturation on nonverbal neuropsychological test performance among latino/a patients with Epilepsy. *App. Neuropsychol.* 21 108–119. 10.1080/09084282.2013.768996 24826504

[B36] Salinas C. M. Bordes-Edgar V. Puente A. E. (2016). “Barriers and practical approaches to neuropsychological assessment of spanish speakers,” in *Minority and Cross-Cultural Aspects of Neuropsychological Assessment: Enduring and Emerging Trends*, 2nd Edn, ed. Ferraro F. R. (New York, NY: Taylor & Francis), 229–258.

[B37] Siedlecki K. L. Manly J. J. Brickman A. M. Schupf N. Tang M.-X. Stern Y. (2010). Do neuropsychological tests have the same meaning in Spanish speakers as they do in English speakers? *Neuropsychology* 24 402–411. 10.1037/a0017515 20438217 PMC3908788

[B38] Steinberg L. Thissen D. (2006). Using effect sizes for research reporting: examples using item response theory to analyze differential item functioning. *Psychol. Methods* 11 402–415. 10.1037/1082-989X.11.4.402 17154754

[B39] Teresi J. A. (2006). Different approaches to differential item functioning in health applications: advantages. disadvantages and some neglected topics. *Med. Care* 44(Suppl. 3), S152–S170. 10.1097/01.mlr.0000245142.74628.ab 17060822

[B40] Uzzell B. P. Ponton M. Ardila A. (2013). *International Handbook of Cross-Cultural Neuropsychology.* Hoboken: Taylor and Francis.

[B41] Zimmerman M. E. Pan J. W. Hetherington H. P. Katz M. J. Verghese J. Buschke H. (2008). Hippocampal neurochemistry, neuromorphometry, and verbal memory in nondemented older adults. *Neurology* 70 1594–1600. 10.1212/01.wnl.0000306314.77311.be 18367703 PMC2735229

